# Influence of Different Types of Corticosteroids on Heart Rate Variability of Individuals with Duchenne Muscular Dystrophy—A Pilot Cross Sectional Study

**DOI:** 10.3390/life11080752

**Published:** 2021-07-27

**Authors:** Rodrigo Martins Dias, Rosangela Akemi Hoshi, Luiz Carlos Marques Vanderlei, Carlos Bandeira de Mello Monteiro, Mayra Priscila Boscolo Alvarez, Tânia Brusque Crocetta, Luis Fernando Grossklauss, Deborah Cristina Gonçalves Luiz Fernani, Maria Tereza Artero Prado Dantas, Fabiana Paula Almeida Martins, David M. Garner, Luiz Carlos Abreu, Celso Ferreira, Talita Dias da Silva

**Affiliations:** 1 Postgraduate Program in Medicine (Cardiology) at Paulista School of Medicine, Federal University of São Paulo (EPM/UNIFESP), São Paulo 04024-002, Brazil; ferreira.celso@unifesp.br (C.F.); talita.dias@unifesp.br (T.D.d.S.); 2 Cardiology Department, Brigham and Women’s Hospital, Boston, MA 02115, USA; Rhoshi@bwh.harvard.edu; 3 Department of Physiotherapy, Faculty of Science and Technology, FCT/UNESP, Presidente Prudente 19060-900, Brazil; lcm.vanderlei@unesp.br; 4 Postgraduate Program in Rehabilitation Sciences, Faculty of Medicine, University of São Paulo (FMUSP), São Paulo 05360-160, Brazil; carlosmonteiro@usp.br (C.B.d.M.M.); mayra.alvarez@educadores.net.br (M.P.B.A.); 5 Department of Health Sciences, Anhanguera College- Campus of Jundiaí, Jundiaí 13209-355, Brazil; 6 Laboratório de Psicologia do Esporte e do Exercício, Centro de Ciências da Saúde e do Esporte, Universidade do Estado de Santa Catarina, Florianópolis 88035-001, Brazil; tania.crocetta@udesc.br; 7 Department of Neurology/Neurosurgery, Neuropediatrist at the Federal University of São Paulo, São Paulo 04039-002, Brazil; lf.grossklauss@unifesp.br; 8 Department of Health Sciences, University of Western Paulista (UNOESTE), Presidente Prudente 19050-920, Brazil; deborah@unoeste.br (D.C.G.L.F.); mariatereza@unoeste.br (M.T.A.P.D.); 9 Study Design and Scientific Writing Laboratory, ABC Medical School (FMABC), Santo André 09060-870, Brazil; fabianapamartins@fmabc.br; 10 Cardiorespiratory Research Group, Department of Biological and Medical Sciences, Faculty of Health and Life Sciences, Headington Campus, Oxford Brookes University, Gipsy Lane, Oxford OX3 0BP, UK; dgarner@brookes.ac.uk; 11 Department of Integrated Health Education, Federal University of Espírito Santo (UFES), Vitória 29040-090, Brazil; abreu.luizcarlos@ufes.br; 12 Faculty of Medicine, University of Sao Paulo City (UNICID), São Paulo 03071-000, Brazil

**Keywords:** Duchenne Muscular Dystrophy (DMD), autonomic nervous system, Heart Rate Variability (HRV), deflazacort, prednisone

## Abstract

Individuals with Duchenne Muscular Dystrophy (DMD) have an impairment of cardiac autonomic function categorized by parasympathetic reduction and sympathetic predominance. The objective of this study was to assess the cardiac autonomic modulation of individuals with DMD undergoing therapy with Prednisone/Prednisolone and Deflazacort and compare with individuals with DMD without the use of these medications and a typically developed control group. Methods: A cross-sectional study was completed, wherein 40 boys were evaluated. The four treatment groups were: Deflazacort; Prednisone/Prednisolone; no corticoid use; and typical development. Heart Rate Variability (HRV) was investigated via linear indices (Time Domain and Frequency Domain) and non-linear indices Results: The results of this study revealed that individuals with DMD undertaking pharmacotherapies with Prednisolone demonstrated HRV comparable to the Control Typically Developed (CTD) group. In contrast, individuals with DMD undergoing pharmacotherapies with Deflazacort achieved lower HRV, akin to individuals with DMD without any medications, as demonstrated in the metrics: RMSSD; LF (n.u.), HF (n.u.), LF/HF; SD1, α1, and α1/α2, and a significant effect for SD1/SD2; %DET and Ratio; Shannon Entropy, 0 V%, 2 LV% and 2 ULV%. Conclusions: Corticosteroids have the potential to affect the cardiac autonomic modulation in adolescents with DMD. The use of Prednisone/Prednisolone appears to promote improved responses in terms of sympathovagal activity as opposed to Deflazacort.

## 1. Introduction

Duchenne muscular dystrophy (DMD) is a hereditary disease, genetically recessive, formed by a mutation of the dystrophin gene [1] and positioned on the Xp21 chromosome [2]. It has an incidence of about 5000 cases in boys per year and its signs and symptoms begin in early childhood [3]. Although respiratory failure is the principal cause of death in these patients, with the advancement of respiratory support technology, cardiac disorders are becoming an important issue [4].

Individuals with DMD have significant impairment of cardiac autonomic function characterized by parasympathetic reduction and sympathetic predominance, which becomes more prominent as the disease progresses with age [5]. It is normal that, once the muscle fibrosis increases in proportion to age, there is an inflammation process occurring continuously throughout their bodies.

It is recognized that in the presence of injury to the human body (for instance muscle necrosis), a reflexive and localized response initiates an inflammatory process that triggers the brain to eliminate the threat via the autonomic nervous system (ANS) with an efferent signal through vagus-inhibiting pro-inflammatory cytokine synthesis [6]. The heart rate variability (HRV) is defined as the unpredictability between heart beats and can be separated into various indices of measurement, reflecting ANS influence on cardiac control [7,8]. Furthermore, the indices that reflect the parasympathetic branch of ANS are reduced with the onset of an inflammation process, such as muscle fibrosis in people with DMD, corroborating the position that the sympathetic branch is modulating even during rest periods, and it is related to cardiac events [9] and sudden death [10].

Thus, corticosteroid treatment has been enforced with the objective of promoting muscle regeneration and differentiation, besides anti-inflammatory effects in the short term [11]. Guidelines for the treatment of DMD now mention application of corticosteroid before the beginning of motor deterioration. The two main corticosteroids prescribed in the treatment of DMD are Prednisone/Prednisolone and Deflazacort [12].

Despite the expected properties of these pharmacotherapies, fat mass gain, endocrine, and bone and behavioral disorders are significant side effects affiliated with the prolonged use of corticosteroids [11,13]. Little is known about the influence of corticosteroids on cardiac autonomic modulation [14,15]. This proves the importance of novel studies related to the possible influence of corticosteroids on cardiac autonomic modulation and its possible effects on the pathologies that cause a chronic process of inflammation, as in DMD.

Taking into consideration the anti-inflammatory effects of corticosteroids and the influence they may promote on the ANS, we hypothesized that their use could enhance cardiac autonomic modulation in individuals with DMD compared with individuals with DMD but without these medications. Consequently, the study objective was to assess the cardiac autonomic modulation of individuals with DMD undergoing therapy with Prednisone/Prednisolone and Deflazacort, and compare this with individuals with DMD without use of these medications and a typically developed control group.

## 2. Materials and Methods

### 2.1. Ethical Aspects

The research project was submitted to the Research Ethics Committee of the Federal University of São Paulo and was approved with CAAE number: 09942913.4.0000.5505. To participate in the research, the Free and Informed Consent Term was signed by the legal guardian of each research subject and the Assent Form by the research subjects aged 17 years or younger. This study was registered on Clinical trials: NCT04740554.

### 2.2. Study Type, Location and Population

A cross-sectional study was completed wherein 40 boys, aged 11 to 18 years, were evaluated. The recruitment of groups was accomplished at the neuromuscular disease outpatient clinic of the Federal University of São Paulo (UNIFESP).

The individuals were split into four groups according to the use and type of corticoid medication used: DMD that enforced Deflazacort (DMD-D), with n = 11; DMD that enforced Prednisone/Prednisolone (DMD-P), with n = 9; DMD Control without corticoid use (DMD-C), with n = 10; and Controls with typical development (CTD), with n = 10. The procedure was commenced during the evaluation that was completed at outpatient follow-up.

### 2.3. Search Inclusion and Exclusion Criteria

All patients diagnosed with DMD confirmed by molecular methods and/or protein expression of skeletal muscle undergoing clinical follow-up at the outpatient clinic for neuromuscular diseases at the Federal University of São Paulo (UNIFESP) were eligible to participate in the study if authorized by their parents or guardians.

We excluded patients with cardiac arrhythmias and atrioventricular blocks, congenital anomalies such as congenital heart defects, pulmonary deformity, and patients using pharmacotherapies that deter ANS, such as antiarrhythmic agents and medications for the treatment of diabetes mellitus, e.g., insulin.

### 2.4. Sample Characteristics

#### 2.4.1. Functional Variables

Anthropometry was measured in all participants and functionality was assessed in DMD patients using Vignos scales and motor function measure (MFM). The Vignos scale is a rating scale for lower limbs, graded 1 to 10, and was used to characterize the sample. The MFM involves 32 items (tasks) separated into three domains that provide a detailed profile of physical disability: (D1) transfers and standing posture; (D2) axial and proximal motor capacity; and (D3) distal motor capacity.

#### 2.4.2. Cardiorespiratory Variables

Initially, the resting ECG was investigated; there was no sinus rhythm or presence of arrhythmias and blocks. Resting measurements completed directly before and after cardiac autonomic modulation assessment including systolic blood pressure (SBP) and diastolic blood pressure (DBP), heart rate (HR), Respiratory Rate (RF-to ensure that at the beginning and at the end of collection, RF remains between 9 to 24 rpm, in the region of 0.15 to 0.40 Hz), and partial oxygen saturation (SO2).

Heart rate was logged via the cardio-frequency meter (RS800CX, Polar Electro, Kempele, Finland) and the SO2 by a digital oximeter (DX2010, Dixtal, Manaus, AM, Brazil) connected to the participant’s index finger or hallux, via a sensor of age-appropriate size, in standard laboratory air. Hemoglobin oxygen saturation was charted after the first minute of stabilization, as the value that remains most constant throughout the second minute. Blood pressure was verified indirectly using an aneroid sphygmomanometer (Tycos) situated on the patients’ left arms and a stethoscope over the median area of the antecubital fossa. Respiratory rate (RF) was measured by counting respiratory incursions during a one-minute period.

### 2.5. Heart Rate Variability (HRV)

Heart rate was recorded beat-to-beat via a cardio-frequency meter (Polar RS800CX, Polar Electro, Kempele, Finland) at 1 kHz sampling rate to assess cardiac autonomic modulation. With the chest strap and monitor, the individuals were supinely placed and remained at rest with spontaneous breathing for 25 min. The laboratory temperature was controlled at 20 °C, and data classification was performed in the morning or afternoon, always with a minimum 2 hr fasting. Participants were asked to avoid consuming coffee, chocolate, or alcohol and taking medications the night before.

Heart rate variability was performed on 1000 consecutive RR intervals from the most stable segment of the tachogram. Only series with less than 5% error were considered suitable for analysis. The data were filtered using the Polar Precision Performance software (Polar Electro, Finland) [16] in moderate mode followed by a visual inspection.

HRV was assessed via linear (time and frequency domains and Poincaré Plot quantitative analysis) using the Kubios^®^ HRV software (version 2.2, Kuopio, Finland). We computed the HRV non-linear features through Poincaré plot, Detrended Fluctuation Analysis (DFA), Recurrence plot (RP), and Symbolic analysis (SA).

The DFA exponents α1 and α2 were measured via software developed by Peng et al. (1995), available at www.physionet.org/physiotools/dfa/#software-for-dfa (accessed on 30 January 2020) (Goldberger et al., 2000) [17].

The recurrence graph was created using the Visual Recurrence Analysis software (v. 4.9) provided by E. Kononov [18], obtainable at http://visual-recurrence-analysis.software.informer.com/4.9/ (accessed on 30 January 2020), and the configured parameters were identical to those enforced by Baptista et al. (dimension = 10, delay = 1, radio = 70 e line = 2).

#### 2.5.1. HRV Linear Analyses

##### Time Domain

Time domains were obtained: SDNN (standard deviation of the normal RR intervals recorded in a time interval); and rMSSD (square root of the mean of the square of the differences between adjacent normal RR intervals in a time interval), expressed in ms [19,20,21].

##### Frequency Domain

For the frequency domains, the analysis divided HRV into fundamental oscillatory components, which were, principally [21,22,23,24]: high frequency components in normalized units (HF_nu_) and low frequency components also in normalized units (LF_nu_) [20,21,22]. The algorithm used for spectral analysis was the fast Fourier transform, (Blackman-Tukey, FFT) (256 s window with 50% overlap).

#### 2.5.2. HRV Nonlinear Analysis

##### Poincaré Plot

The quantitative analysis of the Poincaré Plot included the analysis of the SD1, SD2, and SD1/SD2 indices. The indices specified by the plot are founded on the concept of different temporal effects of changes in the vagal and sympathetic modulation of HR in the subsequent RR intervals, which do not require the stationary quality of data [25,26,27].

The Poincaré plot is based on the autonomic changes in heart rate and, by the dispersion of the graph, it is plausible to suggest that there was a high or low HRV [27]. A qualitative analysis of the Poincaré Plot was attained. The qualitative analysis involved the observation of the dispersion of points on the graph: the greater the spread, the higher the variability.

##### Detrended Fluctuation Analysis (DFA)

We evaluated short- and long-term scale exponents: α1 resembled a period of 4 to 11 beats, and α2 matched a period from 11 to 64 beats [28,29]. This analysis demonstrated, in a quantifiable way, the presence or absence of fractal correlation properties of the RR intervals.

##### Recurrence Plot

The recurrence plot breakdown is needed to assess the time dependence of a series, precisely in the assessment of stationarity [30]. The recurrence graph renders it conceivable to visualize the behavior of trajectories in the phase space and, additionally, to offer the periods where the state of a dynamic system is recurring [31,32]. These aspects can be confirmed regarding the quantitative analysis, which presented indices such as: mean; SD; %REC: recurrence rate; %DET: determinism; %LAM: laminarity; TT: trapping time; and ratio.

##### Symbolic Dynamic Analysis

We scrutinized four patterns: 0V for patterns with no variation (all symbols are equal, e.g., 4,4,4 or 2,2,2); 1V was related to patterns with one variation (two consecutive symbols are equal and the remaining symbol is different, e.g., 3,4,4 or 4,4,2); 2LV for patterns with two similar variations between successive symbols (two successive increases or decreases, e.g., 321 or 135); and 2UV for two dissimilar variations between successive symbols (one decrease followed by an increase or the other way around, e.g., 2,4,2 or 4,1,2) [33].

### 2.6. Data Analysis

As dependent variables, we quantified all linear and non-linear HRV indices. Differences between groups (DMD with Deflazacort [DMD-D]-1, DMD with Prednisolone [DMD-P]-2, DMD without pharmacotherapies [DMD-N]-3, and control with typical development [CTD]-4) were studied with the Multivariate Analysis of Variance (MANOVA). Least significant difference (LSD) post hoc tests were finalized on each pair of groups. Partial eta-squared (ŋ_p_^2^) was stated to measure effect size and was interpreted as small (between >0.01 and ≤0.06), medium (>0.06 and ≤0.14), or large (>0.14) [34], and we similarly described observed power (op). Results were significant at the level *p* < 0.05 (or, <5%). Statistical analyses were computed via IBM-SPSS (v. 26.0, IBM Corp., Armonk, NY, USA).

## 3. Results

### 3.1. Baseline Characteristics

Table 1 summarizes the clinical information of DMD subjects and the control group.

### 3.2. Linear Index of Time Domain and Frequency Domain

For Time and Frequency domains (Table 2), the MANOVA could not establish a significant effect between groups (Wilks Lambda = 0.049, F60, 45 = 1.32, *p* = 0.160, ŋp2 = 0.63, op = 0.93). When observing the separate ANOVAs, there were significant effects for mean RR (F3, 34 = 8.06, *p* < 0.001, ŋp2 = 0.42, op= 0.98), RMSSD (F3, 34 = 3.13, *p* = 0.038, ŋp2 = 0.22, op = 0.68), LF (n.u.) (F3, 34 = 4.87, *p* = 0.006, ŋp2 = 0.30, op = 0.87), HF (n.u.) (F3, 34 = 5.04, *p* = 0.005, ŋp2 = 0.31, op = 0.88), and LF/HF (F3, 34 = 4.31, *p* = 0.011, ŋp2 = 0.28, op = 0.82).

### 3.3. Non-Linear Index

#### 3.3.1. Poincaré Plot and DFA

There were significant effects for SD1 (F3, 34 = 3.15, *p* = 0.037, ŋp2 = 0.22, op = 0.68), α1 (F3, 34 = 3.76, *p* = 0.020, ŋp2 = 0.25, op = 0.76), and α1/ α2 (F3, 34 = 3.73, *p* = 0.020, ŋp2 = 0.25, op = 0.76), and a moderately significant effect for SD1/SD2 (F3, 34 = 2.75, *p* = 0.058, ŋp2 = 0.20, op = 0.61). The values of central tendency and distributions are illustrated in Table 3, in addition to the post-hoc analysis.

**Figure 1:**
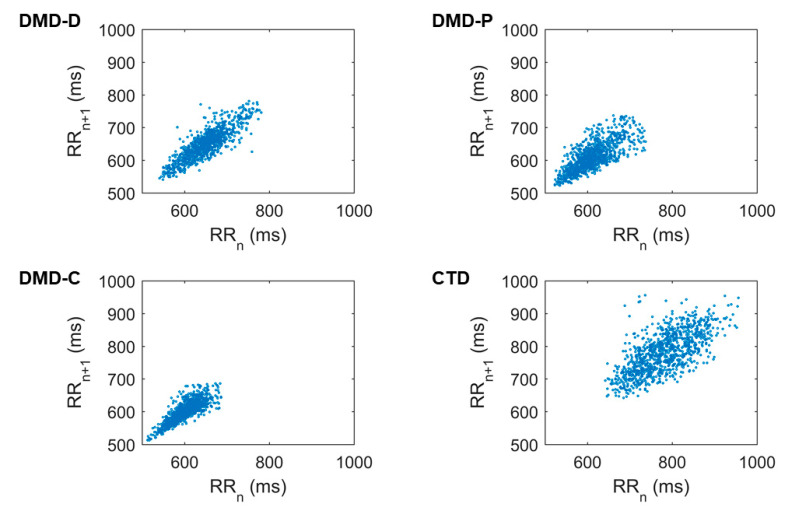
Poincaré Plot. DMD-D, Duchenne Muscular Dystrophy-Deflazacort; DMD-P, Duchenne Muscular Dystrophy-Prednisone/Prednisolone; DMD-C, Duchenne Muscular Dystrophy–no corticosteroids; and CTD, Control typical development.

Visual analysis exposed changed dispersion, of which the DMD-C group presented less dispersion and CTD greater. Amongst the DMD-D and DMD-P groups, greater dispersion was perceived in the DMD-P group, a situation that indicated an improved HRV, and reduction in the DMD-D group (Figure 1).

#### 3.3.2. Recurrence Plot Analysis

**Figure 2:**
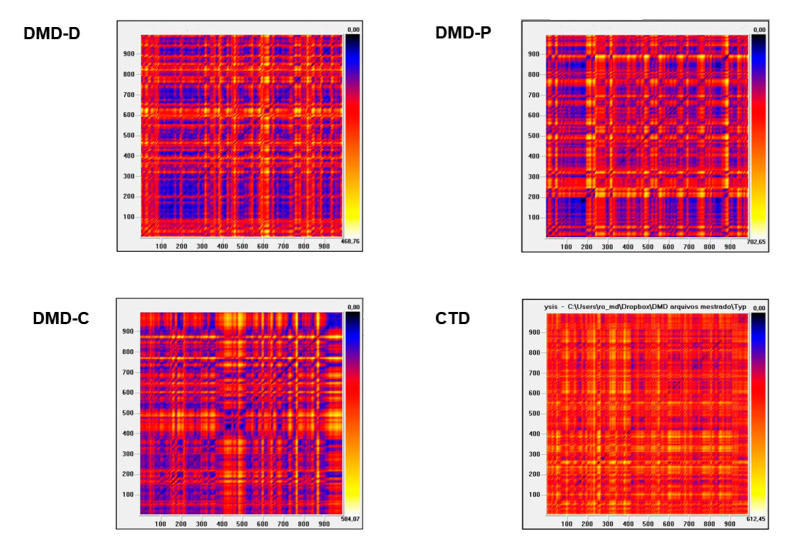
Recurrence Plot. DMD-D, Duchenne Muscular Dystrophy-Deflazacort; DMD-P, Duchenne Muscular Dystrophy-Prednisone/Prednisolone; DMD-C, Duchenne Muscular Dystrophy–No corticosteroids; and CTD, Control typical development.

The Recurrence Plot (Figure 2) qualitative analysis confirmed the DMD-D and DMD-C groups had extra response patterns, indicated by the large number of blue and black dots and more square shapes, an element that indicates a lower HRV in these individuals. The DMD-P group similarly expressed patterns with a considerable number of blue dots, but in a lesser capacity when compared with the DMD-D and DMD-C groups; additionally, the pattern exposed had fewer squares, suggesting a higher HRV. The graph pattern of the CTD group was the one that indicated the optimal HRV amongst the four groups.

MANOVA did not detect significant effects between groups (Wilks Lambda = 0.42, F27, 76 = 0.98, *p* = 0.530, ŋp2 = 0.25, op = 0.71). However, the separate ANOVAs revealed significant effects on mean RR (F3, 34 = 3.57, *p* = 0.024, ŋp2 = 0.24, op = 0.74), %DET (F3, 34 = 3.73, *p* = 0.020, ŋp2 = 0.25, op = 0.76) and ratio (F3, 34 = 3.38, *p* = 0.029, ŋp2 = 0.23, op = 0.72). The central tendency values and distributions are illustrated in Table 3, as well as the post-hoc analysis.

#### 3.3.3. Symbolic Analysis

MANOVA revealed significant effects between groups (Wilks Lambda = 0.176, F18, 79 = 3.14, *p* < 0.001, ŋp2 = 0.44, op = 0.99). The separate ANOVAs revealed significant effects for Shannon Entropy (F3, 33 = 3.0, *p* = 0.044, ŋp2 = 0.21, op = 0.65), 0V% (F3, 33 = 4.13, *p* = 0.014, ŋp2 = 0.27, op = 0.80), 2LV% (F3, 33 = 3.37, *p* = 0.030, ŋp2 = 0.23, op = 0.71), and 2ULV% (F3, 33 = 3.98, *p* = 0.016, ŋp2 = 0.27, op = 0.79). The central tendency values and distributions are illustrated in Table 4, as well as the post-hoc analysis.

## 4. Discussion

The main consequence of this study revealed those individuals with DMD undergoing pharmacological treatment with Prednisolone demonstrated HRV comparable to control with typical development (CTD). In contrast, individuals with DMD undergoing pharmacotherapies with Deflazacort showed lower HRV, similar to individuals with DMD without the use of any medications.

Individuals who take Prednisone/Prednisolone have enhanced functional capacity, both in MFM and in the Vignos scale (Table 1). Thus far, we cannot attest to whether this group had a healthier physiological function because of the use of Prednisolone or if the group that used Prednisone/Prednisolone had randomly better functionality. We conclude that these results warrant further investigation in future research as the results in the scientific literature regarding these aspects are inconsistent [35,36].

This response may be, in any case, related in part to the likely influence of corticosteroids on vagal activity [20]. This aspect can be observed by the higher values of HRV indices that modulate the parasympathetic action (RMSSD, SD1, HF (n.u.), (2 LV% and 2 ULV%) in the group using Prednisone/Prednisolone. The symbolic analysis equally demonstrated that, in addition to the reduction in parasympathetic activity, there was likewise a predominance of sympathetic activity, a situation that can be indicated by the 0V% indices, which were increased in DMD-D and DMD-C when compared with DMD-P and CTD [21,37,38]; 0% represents cardiac sympathetic modulation [39].

Moreover, higher overall variability was detected by the indices SDNN and SD2 (significantly higher in DMD-C group vs CTD group). This indicator might be related to the better parasympathetic modulation, as the HF index revealed (significantly lower in the DMD-D and DMD-C groups when compared with the DMD-P and CTD groups).

The responses designated above were also detected by the qualitative analysis of the Poincaré plot, which displayed superior dispersion of points on the DMD-P graph when compared with DMD-D and DMD-C; the CTD group showed the greatest dispersion of all four. This analysis highlighted an improved autonomic modulation in the group that administers Prednisone/Prednisolone when compared with the groups that have Deflazacort and DMD-C.

The autonomic imbalance between SNP and SNS in these subjects was revealed by other researchers [40]. Nevertheless, our results indicate that the usage of Prednisone/Prednisolone in adolescents with DMD promotes increases in HRV of similar values in their typically developed counterparts, and this is very clinically relevant.

Reduction of HRV is related to an increased risk of morbidity and mortality from many causes and the progression of several risk factors [41]. In DMD, the tendency is the development of heart disease by reason of morpho-functional changes already shaped by the clinical condition in these individuals [42]. Thus, a better autonomic modulation of the DMD-P group compared with the DMD-D and DMD-C groups may be connected to greater cardiac protection.

These overall results might establish the beneficial influence of the use of corticosteroids on the ANS of adolescents with DMD, mostly of Prednisolone, which revealed the greatest number of values of the HRV indices to be analogous with the typical developed control group.

These results are in proportion to Golczynska, et al. [43], who recognized that the Prednisone significantly reduced sympathetic nerve activity in the normal subjects.

This point could be related to the conceivable crossing of endogenous and synthetic corticosteroids via the blood-brain barrier and binding to adrenergic and noradrenergic nerve cells, as it has been shown that adrenergic and noradrenergic nerve cells offer solid immunoreactivity to the corticosteroid receptors in rat brains [44]. As well as these data revealing the influence of corticosteroids on the ANS when associating DMD with corticosteroids to DMD without these pharmacotherapies and to typically developed controls, they similarly revealed a difference between these medications on HRV of adolescents with DMD.

This change between Prednisolone and Deflazacort may be similar to the effects of the use of Prednisolone on both skeletal and cardiac muscle tissue, which are formulated of complex protein networks responsible for the transmission of the stimulus and contraction. In their study, Gaud, et al. [45] displayed that prednisone reduced the number of degenerated cells in the *C. elegans* model by 40% and that it was not prone to inflammation. It was suggested that prednisone has a direct effect on muscle tissue survival. In skeletal muscle tissue, Ohlendieck, et al. [46] studied the status of dystrophin-associated proteins in the muscle of 17 DMD patients of many age groups; the results exposed a dramatic reduction in all dystrophin-associated proteins in the sarcolemma of the DMD muscle compared with normal muscle and the muscle of a variety of other neuromuscular diseases. This abnormality was detected in all 17 DMD patients regardless of age, a fact that suggests that the lack of dystrophin leads to the loss of all proteins linked with dystrophin.

In cardiac muscle cells, dystrophin has an important role, identically to skeletal muscle, i.e., the lack of dystrophin can cause an increase in the structural vulnerability of cardiac muscle cells, membrane instability, interruption of Ca^2+^ balance, enlarged production of reactive oxygen species, and mitochondrial dysfunction [47]. In the heart of DMD subjects, the deficiency in the production of dystrophin, not only in the cardiac muscle but similarly in the endothelial cells, in the vascular smooth muscle, and in the fibroblasts, causes impairments in the cardiac functionality [48,49,50,51]. It is notable that, due to the deficiency in dystrophin production, the functioning of blood vessels is dysfunctional [49], a state that causes the reduction of the muscles’ vascular network [48,50].

Muscle damage (e.g., cardiac, skeletal, and smooth) is progressive and for its treatment, the use of glucocorticoids is suggested. Glucocorticoids act as anti-inflammatories and are often applied to prevent progressive muscle damage, but they have many adverse events, which can promote weight gain, osteopenia and osteoporosis, diabetes, and muscle atrophy [52]. Prednisone/prednisolone and Deflazacort act predominantly by inhibiting NF-κB signaling, a function that has shown long-term protective effects and serves as the gold standard for the treatment of DMD [53,54] to mitigate damage to both skeletal and cardiac muscle.

In their study, Peterson JM, et al. [55] indicated that NF-κB inhibition in mdx mice improves cardiac function. The results suggested that the mdx heart muscle had an impaired ability to relax and was therefore unable to respond to β-adrenergic stress with an accelerated heart rate. The authors also indicated that pharmacological inhibition of NF-κB for just 1 month can reverse existing stress-induced cardiac muscle dysfunction and that inhibition of NF-κB systemically and specifically in cardiomyocytes reverses the pathological disability of cardiac muscle preparations for responding to β-adrenergic stimulation. As stated by the authors, these results demonstrated that the NF-κB of cardiomyocytes impairs the cardiac response to β-adrenergic stress, hence providing the first indication that the classic NF-κB signaling derived from cardiomyocytes plays an active role in the promotion of dystrophic cardiac dysfunction. It is recognized that the activation of NF-κB provides a reduction in the anti-inflammatory mechanism that is related to β-adrenergic agonists. The main effect of β-agonists is linked to the interaction with β-adrenergic receptors, followed by the activation of calcium-dependent protein kinases. Activation of NF-κB also represses calcium genes. Interruption of calcium homeostasis is considered to cause the pathology of dystrophic hearts [55,56].

This detail establishes that the inhibition of NF-κB may be connected to the improvement of β-adrenergic responses and, hence, an improved cardiovascular autonomic response. This verifies our data, which displayed an improvement in HRV in individuals with DMD who take corticosteroid therapies, with an importance on the prescription of Prednisone/Prednisolone.

DFA assessment serves as a metric for the presence or absence of fractal correlation properties of RR intervals by time series data. As stated by Acharya and Joseph [57], α1 should be close to unity, and α2 should always be less than α1, in physiologically healthy individuals. The α1 exponent of the short-term fractal scale assessed in DFA can be enforced to predict fatal cardiovascular events in numerous situations. α1 close to 0.5 displays no correlation between the values, and is considered as white noise, or a random signal. Still, α1 approximating to unity are features of processes termed fractals, which are associated with dynamic behavior of generated time series by complex systems, for instance, the autonomic regulation of a healthy subjects’ sinus rhythm [35,36] and signified by the 1/f noise, akin to the stability and adaptability of healthy physiological and complex systems. α ≥ 1 is referred to as Brownian noise and as such is a non-stochastic long-distance correlation [58,59,60], that is, a process that has a predictable way to evolve. In our data, this pattern was recurring. In the group of individuals with DMD taking Prednisone/Prednisolone and also developed controls, α1 was just below 1, which represents a more balanced autonomic regulation, and α2 < α1. In the (DMD-D) and (DMD-C) groups, α1 > 1 and α2 < α1.

DFA also supports the Recurrence plot findings, as the indices are also correlated to the complexity of cardiac autonomic modulation. According to Webber et al. [61], high %REC values are connected to periodic systems compared with non-periodic, whereas high %DET values are correlated to structured and deterministic systems, namely, high %REC and %DET values indicate reduced responses of the cardiac autonomic modulation complexity. However, when deliberating Recurrence Plot indices such as TT and %LAM, the lower the values, the higher the complex response of the system. Our data indicated reduced complexity and a correspondingly reduced chaotic system response for the cohort that took Deflazacort and greater complexity and chaotic response in the systems in those subjects that used Prednisone/Prednisolone.

The qualitative (or visual) analysis of the recurrence plot displayed different complexities amongst the groups. In this study it was documented that the DMD-D and DMD-C groups had more points of the same configuration status (blue and black), but in the DMD-P group, while having a greater number of these points, they were in a smaller quantity, this being more similar to the CTD group, which represents the group with the highest number of points with different configuration states (orange and white points). This situation proves that individuals with DMD who take Deflazacort or who do not use medications have less system complexity or chaotic response when compared with individuals who take Prednisone/Prednisolone, which is closer to individuals of typical development.

The recurrence plot has clinical significance worth highlighting; a presentation with more geometric patterns could be detected in healthy newborns, the elderly, and sick patients, which Marwan [62] referred to as “large dark rectangles”. Thus, this property indicates less heart rate variability, either as a result of the ANS immaturity or the progressive loss of function.

Along with these, a factor that should be highlighted is Shannon Entropy. This determines the degree of complexity in the distribution of a signal, a detail that permits the identification of conditions that may deter cardiovascular regulation [33].

Cardiovascular difficulties can aggravate morbidities in these individuals and an early diagnosis or a form of prevention is necessary to prevent future damage [42,63]. HRV is an imperative health predictor [21] that can predict cardiovascular risks [21,22]. This demonstrates a need to intermittently assess HRV to monitor the clinical development of the cardiovascular condition of these patients and, accordingly, analyzing the system’s behavior and complexity is interesting. Here, DFA and Recurrence Plot analysis are crucial.

As study limitations, we can highlight the point that this was an observational study, so we suggest that future clinical trials should be completed with the aim of observing causality and effect of the Prednisone/Prednisolone in the ANS of adolescents with DMD. Moreover, the sample size of the present study can be considered a limitation, though we believe that the effect sizes found were large, which attest to the dependability of the results presented. Hence, more robust studies with larger sample sizes are essential to establish if these effects can be replicated. Additionally, the mode of action and the target organ in response to the HRV have not been studied and the age group of DMD is not wide.

## 5. Conclusions

Corticosteroids have the potential to positively influence the cardiac autonomic modulation in adolescents with DMD. The use of Prednisone/Prednisolone appeared to induce improved responses in terms of sympathovagal activity as opposed to Deflazacort.

## Figures and Tables

**Table 1:** Characteristics and clinical features.

	DMD-D	DMD-P	DMD-N	CTD	*p*-Value
Age	15.3 ± 2.0	14.1 ± 1.7	14.9 ± 1.6	14.7 ± 2.3	0.355
BMI	21.9 ± 4.4	19.3 ± 4.4	22.8 ± 6.2	19.5 ± 3.2	0.329
FUNCTIONAL SCALES					
MFM-total	43.1 ± 12.5	64.5 ± 25.1 ^a,b^	45.4 ± 13.9	-	0.025
Vignos	7.3 ± 0.5	4.9 ± 2.4 ^a,b^	6.80 ± 1.4	-	0.004
CARDIOVASCULAR INFORMATION					
SBP i	100.45 ± 10.04	96.78 ± 5.69	99.9 ± 10.23	-	0.861
SBP f	98.45 ± 12.77	99.33 ± 13.37	100.3 ± 11.49	-	
DBP i	64.27 ± 10.81	58.44 ± 7.98	62.1 ± 9.45	-	0.581
DBP f	65.55 ± 10.78	60.33 ± 5.14	60.5 ± 10.38	-	
HR i	92.27 ± 15.4	90.44 ± 8.74	97.9 ± 8.97	-	0.460
HR f	92.73 ± 14.33	94.67 ± 18.73	97.2 ± 10.44	-	
RR i	21.45 ± 4.56 ^c^	22 ± 3.1	20 ± 3.6	-	0.042 *
RR f	18.73 ± 3.8	20.89 ± 3.48	19.8 ± 1.9	-	
SatO2 i	97.64 ± 0.9	97.67 ± 0.70	97.7 ± 0.48	-	0.670
SatO2 f	97.82 ± 0.04	97.22 ± 1.09	97.8 ± 0.42	-	

Legend: DMD-D, Duchenne Muscular Dystrophy-Deflazacort; DMD-P, Duchenne Muscular Dystrophy-Prednisone/Prednisolone; DMD-C, Duchenne Muscular Dystrophy-No corticosteroids; and CTD, Control typical development; BMI, Body Mass Index; SBP, Systolic Blood Pressure; DBP, Diastolic Blood Pressure; HR, Heart Rate; RR, R-R Interval; SatO2, O2 saturation; i-initial; f, final; ^a^ significant difference between DMD-D and DMD-P; ^b^ significant difference between DMD-D and DMD-C; ^c^ difference between initial and final moments; * post hoc test showed that the difference between initial and final RR (*p* = 0.014) occurred only in group DMD-D. Control typically developed group values were missing; b-before, a-after.

**Table 2:** HRV Indices.

		(1) DMD-D, *n* = 11	(2) DMD-P, *n* = 9	(3) DMD-C, *n* = 10	(4) CTD, *n* = 10	Post-Hoc Tests (LSD)					
						(1) × (2)	(1) × (3)	(1) × (4)	(2) × (3)	(2) × (4)	(3) × (4)
Mean RR	Mean ± SD	657.4 ± 91.4	640.6 ± 46.6	614.6 ± 54.5	761.5 ± 95.4	-	-	0.002	-	0.001	<0.001
	Median (CI)	662.5 (572.7; 711.7)	612 (598.5; 688.0)	601 (567.5; 663.5)	734 (692; 857)						
	% CV	13.9	7.3	8.9	12.8						
SDNN	Mean ± SD	45.2 ± 20.6	48.5 ± 16.5	37.4 ± 13.3	59.4 ± 10.5	-	-	-	-	-	0.019
	Median (CI)	43 (26; 62)	44 (30.5; 63)	32 (26; 49)	63 (47.5; 68)						
	% CV	45.7	34.2	35.5	25.4						
RMSSD	Mean ± SD	28.3 ± 17	36.0 ± 14.8	21.9 ± 10.1	39.8 ± 11.5	-	-	0.077 *	0.035	-	0.008
	Median (CI)	26.0 (4; 7.2)	31.0 (21.5; 52)	19.0 (13; 31)	38 (30.5; 51)						
	% CV	60.2	41.3	46.5	27.9						
LF_nu	Mean ± SD	69.5 ± 8.6	59.6 ± 5.1	71.3 ± 7.3	62.3 ± 10.1	0.012	-	0.028	0.004	-	0.010
	Median (CI)	66.5 (63.5; 77.7)	59.0 (55.5;63)	70.0 (66; 77)	64 (53.5; 71)						
	% CV	12.4	8.6	10.3	16.9						
HF_nu	Mean ± SD	30.3 ± 8.3	40.4 ± 5.1	28.7± 7.3	37.6± 10.1	0.10	-	0.023	0.004	-	0.010
	Median (CI)	33.5 (22.2; 36.2)	41.0 (37; 44.5)	30.0 (23; 34)	36 (29; 46.5)						
	% CV	27.7	12.7	25.6	26.5						
LF/HF	Mean ± SD	2.8 ± 1.8	1.4 ± 0.5	2.8 ± 1.2	1.8 ± 0.7	0.016	-	0.079*	0.004	-	0.022
	Median (CI)	2.0 (1; 2.5)	1.0 (1;2)	2.0 (2; 4)	2.0 (1; 2.5)						
	% CV	45.2	36.6	43.9	43.8						
SD1	Mean ± SD	20.0 ± 12.1	25.3 ± 10.5	15.4 ± 7.2	28.3 ± 8.1	0.073 *	-	-	0.037	-	0.008
	Median (CI)	18.0 (8;30)	22.0 (15; 36.5)	13.0 (9; 22)	27 (21.5; 36)						
	% CV	60.7	41.6	46.9	27.7						
SD2	Mean ± SD	60.8 ± 27.1	63.7 ± 21.4	50.6 ± 17.3	79.1 ± 13.2	-	-	-	-	-	0.025
	Median (CI)	58.0 (31.7; 82.2)	59.0 (40.5; 80.5)	43.0 (35.5; 66)	83 (64.5; 89)						
	% CV	44.6	33.7	34.4	26.9						
SD1/SD2	Mean ± SD	0.3 ± 0.07	0.4 ± 0.05	0.3 ± 0.06	0.4 ± 0.06	-	-	0.052 *	0.053 *	-	0.027
	Median (CI)	0.3 (0.2; 0.3)	0.4 (0.3; 0.4)	0.3 (0.2; 0.3)	0.3 (0.3; 0.4)						
	% CV	22.6	14.5	20.7	41.4						
α1_DFA	Mean ± SD	1.06 ± 0.13	0.85 ± 0.11	1.03 ± 0.17	0.98 ± 0.11	0.004	-	0.078 *	0.017	-	-
	Median (CI)	1.07 (1.02; 1.14)	0.86 (0.74; 0.92)	0.95 (0.89; 1.19)	1.02 (0.87; 1.07)						
	% CV	12.2	13.1	16.9	17.9						
α2_DFA	Mean ± SD	0.77 ± 0.08	0.78 ± 0.10	0.83 ± 0.08	0.85 ± 0.09	-	-	0.051 *	-	-	-
	Median (CI)	0.74 (0.70; 0.86)	0.79 (0.70; 0.88)	0.83 (0.79; 0.89)	0.83 (0.79; 0.94)						
	% CV	11.6	13.6	10.1	10.2						
α1α2	Mean ± SD	1.4 ± 0.2	1.1 ± 0.2	1.2 ± 0.2	1.1 ± 0.2	0.008	-	0.007	-	-	-
	Median (CI)	1.4 (1.2; 1.5)	1.1 (0.9; 1.2)	1.3 (1; 1.4)	1.2 (1; 1.3)						
	% CV	14.2	19.1	18.4	19.9						

Legend: * marginally significant results. DMD-D, Duchenne Muscular Dystrophy-Deflazacort; DMD-P, Duchenne Muscular Dystrophy-Prednisone/Prednisolone; DMD-C, Duchenne Muscular Dystrophy–No corticosteroids; and CTD, Control typical development; M: Mean; Md: Median; CV: Coefficient of Variation; CI: Confidence Interval; RR intervals: intervals between heart beats; SDNN: standard deviation of normal to normal RR interval; RMSSD: the square root of the mean squared differences of successive normal to normal intervals; LF: low frequency; HF: high frequency; LF/HF: low frequency and high frequency ratio; n.u.: normalized units.

**Table 3:** Visual Recurrence Analysis.

GROUPS						Post-Hoc Tests (LSD)					
**VARIABLES**		**(1) DMD-D**	**(2) DMD-P**	**(3) DMD-C**	**(4) CTD**	**(1) × (2)**	**(1) × (3)**	**(1) × (4)**	**(2) × (3)**	**(2) × (4)**	**(3) × (4)**
		*n* = 11	*n* = 9	*n* = 10	*n* = 10						
%REC	Mean ± SD	26.7 ± 4.5	21.7 ± 4.0	27.9 ± 3.9	24.3 ± 5.2	0.022	-	-	0.006	-	0.057 *
	Median (CI)	25.9(22; 30.4)	22.6(18.4; 24.9)	27.5(24.3; 32.2)	26.2(19.1; 28.9)						
	% CV	17.0	18.7	14.2	21.1						
%DET	Mean ± SD	98.1 ± 0.93	96.9 ± 1.2	98.3 ± 0.8	97.6 ± 1.1	0.016	-	-	0.006	-	0.054 *
	Median (CI)	98.3(97.3; 98.9)	96.9(96; 97.7)	98.3(97.7; 99.1)	97.7(96.5; 98.4)						
	% CV	1.0	1.2	0.8	1.3						
%LAM	Mean ± SD	96.2 ± 2.6	91.6 ± 6.3	97 ± 1.72	94 ± 4.5	-	-	-	-	-	0.068 *
	Median (CI)	97(93.7; 98.3)	93.5(88.6; 95.7)	96.4(95.6; 98.8)	95.2(91.4; 97)						
	% CV	2.7	6.8		19.2						
TT	Mean ± SD	16.5 ± 29.2	5.3 ± 1.6	8.4 ± 0.5	6.6 ± 2.2	-	-	-	-	-	-
	Median (CI)	7(5.7; 10)	5.3(4; 6.7)	7.8(6.3; 10.9)	6.5(4.4; 8.4)						
	% CV	177.1	30.3		36.5						
Ratio	Mean ± SD	3.7 ± 0.6	4.5 ± 0.9	3.6 ± 0.5	4.2 ± 0.9	0.023	0.007	-	-	-	0.065 *
	Median (CI)	3.8(3.2; 4.4)	4.3(3.9; 5.3)	3.5(3;4)	3.7(3.4; 5)						
	% CV	16.1	20.0		22.3						

Legenda: * Marginally significant results. DMD-D, Duchenne Muscular Dystrophy-Deflazacort; DMD-P, Duchenne Muscular Dystrophy-Prednisone/Prednisolone; DMD-C, Duchenne Muscular Dystrophy-No corticosteroids; and CTD, Control typical development; %REC: recurrence rate; %DET: determinism; %LAM: laminarity; TT: trapping time; Ratio; CV: Coefficient of Variation; CI: Confidence Interval; SD: Standard deviation DF: Degree of freedom.

**Table 4:** Symbolic Analysis.

GROUPS						LSD Pairwise Comparison Tests					
**VARIABLES**		**(1) DMD-D**	**(2) DMD-P**	**(3) DMD-C**	**(4) CTD**	**(1) × (2)**	**(1) × (3)**	**(1) × (4)**	**(2) × (3)**	**(2) × (4)**	**(3) × (4)**
		*n* = 11	*n* = 9	*n* = 10	*n* = 10						
ShanEntr	Mean ± SD	3.53 ± 0.33	3.86 ± 0.32	3.48 ± 0.34	3.80 ± 0.32	0.036	-	-	0.021	-	0.052 *
	Median (CI)	3.62(3.3; 3.77)	3.78(3.60; 4.14)	3.5(3.29; 3.7)	3.90(3.40; 4.09)						
	% CV	9.5	8.3	9.9	12.8						
0V%	Mean ± SD	32.59 ± 10.80	22.43 ± 8.32	32.27 ± 9.56	23.15 ± 9.85	0.029	-	0.042	0.008	-	0.012
	Median (CI)	28.96(22.52; 40.96)	23.45(15.73; 28.81)	32.77(27.85; 42.54)	22.48(15.43; 31.54)						
	% CV	33.1	37.1	27.1	39.8						
1V%	Mean ± SD	45.64 ± 4.32	47.66 ± 2.19	46.24 ± 3.80	48.50 ± 4.22	-	-	-	-	-	-
	Median (CI)	47.15(42.58; 48.52)	46.89(45.99; 49.75)	47.6(44.44; 48.55)	48.99(44.96; 51.84)						
	% CV	9.5	4.6	8.2	13.7						
2LV%	Mean ± SD	8.67 ± 4.13	11.83 ± 5.47	6.73 ± 3.15	12.26 ± 4.24	-	-	-	0.018	-	0.011
	Median (CI)	9.32(4.58; 11.12)	11.42(8.42; 13.87)	6.61(4.16; 8.32)	11.40(8.38; 16.10)						
	% CV	47.6	46.3	46.8	34.6						
2ULV%	Mean ± SD	13.08 ± 5.22	18.07 ± 4.75	11.75 ± 3.75	16.06 ± 3.11	0.017	-	-	0.004	-	0.042
	Median (CI)	12.83(8.44; 15.83)	16.03(14.78; 22.49)	13.53(7.16; 14.58)	17.11(13.59; 18.45)						
	% CV	39.9	26.3	32.0	44.8						

Legend: * Marginally significant results. DMD-D, Duchenne Muscular Dystrophy- Deflazacort; DMD-P, Duchenne Muscular Dystrophy-Prednisone/Prednisolone; DMD-C, Duchenne Muscular Dystrophy–No corticosteroids; and CTD, Control typical development; ShanEntr: Shannon Entropy; 0V%: standard without variation; 1V%: standard with 1 variation; 2LV%: standard with two similar variations; 2ULV%: standard with two different variations; CV: Coefficient of Variation; CI: Confidence Interval SD: Standard deviation DF: Degree of freedom.

## Data Availability

The authors accept to make research data available to assist scientific development. It can be obtained from Talita Dias da Silva: write at ft.talitadias@gmail.com.
